# Microarray-Assisted Pathway Analysis Identifies MT1X & NFκB as Mediators of TCRP1-Associated Resistance to Cisplatin in Oral Squamous Cell Carcinoma

**DOI:** 10.1371/journal.pone.0051413

**Published:** 2012-12-10

**Authors:** Bo Peng, Yixue Gu, Yan Xiong, Guopei Zheng, Zhimin He

**Affiliations:** 1 Affiliated Cancer Hospital and Cancer Research Institute, Guangzhou Medical University, Guangzhou, Guangdong, China; 2 Department of Pharmacology, Guangzhou Medical University, Guangzhou, Guangdong, China; 3 Cancer Research Institute, College of Medicine, University of South China, Hengyang, Hunan, China; 4 Cancer Research Institute, Xiangya School of Medicine, Central South University, Changsha, Hunan, China; Virginia Commonwealth University, United States of America

## Abstract

We recently reported that *TCRP1*, a novel multidrug-resistance associated human gene, can mediate cisplatin resistance in OSCC cells. However, the molecular mechanism underlying this role of *TCRP1* remained to be elucidated. In this study, by using Human Toxicology and Drug Resistance Microarray, we identified 30 genes with significantly different expression levels between Tca/PYM and TCRP1 knockdown cell lines. Co-immunoprecipitation experiments and GST-pull down assays showed that metallothionein1X (MT1X) and Akt interact with TCRP1. siRNA-mediated knockdown of TCRP1 and MT1X was found to sensitize cells to cisplatin, leading to increased apoptosis and inhibition of cell proliferation. These functions of TCRP1 may be caused at least in part via activation of the PI3K/Akt/NF-κB signaling pathway. Taken together, our findings indicate that TCRP1 may be an important drug target for improvement of the treatment and survival of patients with oral squamous cell carcinoma.

## Introduction

Platinum-based drugs are currently the largest class of drugs used in treating various types of cancer, such as ovarian, testicular, and head and neck squamous cell carcinomas, including oral squamous cell carcinoma (OSCC) [1], [2], [3]. However, many tumors are completely resistant to these drugs and exhibit no clinical response. Several lines of research have shown that such differences in clinical responses are caused partially by multidrug resistance to these drugs [4]. Although drug efflux mechanisms have been shown to play an important role in the multidrug-resistant phenotype, evidence for additional contributing factors led us to use cDNA microarray to search for differentially expressed genes in the Tca8113 cell line and its pingyangmycin resistant variant (Tca/PYM). Our results revealed a subset of differentially expressed genes in the multidrug resistance model, including a novel gene named *tongue cancer resistance-associated protein 1* (*TCRP1*, Genebank number: EF363480) [5].

In our previous work, we reported that *TCRP1* may be a candidate chemotherapeutic-resistance gene responsible for mediating multidrug resistance in OSCC cells [6]. It is expressed ubiquitously in many cancer cell lines, but has higher expression in cisplatin (DDP)-resistant cell lines [7]. Immunofluorescence and immunohistochemical analyses indicated that *TCRP1* is mainly localized in the cytoplasm, and can promote radio-resistance in OSSC cells [7], [8]. Over-expression of TCRP1 is associated with a poor clinical outcome in cancer patients and is often attributed to resistance to DDP [7]. Meanwhile, transfection of TCRP1 into Tca8113 cells results in a 3.4-fold increase in DDP resistance and enables anchorage-independent growth and colony formation in soft agar [6]. These data suggest that TCRP1 may be a DDP-resistance associated protein. However, the molecular mechanisms underlying *TCRP1*-mediated resistance, such as the target protein for *TCRP1* and the pathways involved in *TCRP1* function in drug resistance remain to be elucidated.

Recently developed techniques for genome-wide expression analysis seek to provide additional information on novel candidate genes associated with *TCRP1*-associated drug resistance and new therapeutic targets [9]. Microarray technology has become a widely used tool to address complex biological questions by simultaneously analyzing changes in the expression of thousands of genes and identifying significant patterns. Identifying patterns of gene expression and grouping genes into expression classes can provide important insights into their biological function and relevance. For example, recent studies have established several DDP-resistant cell models. Microarray technology has led to the discovery of several genes related to DDP resistance, such as *LUM*, *PDE3B*, *CCND1*, and *RECQL*, in OSCC cells [10], [11], [12]. Based on these facts, we reasoned that small interfering RNA (siRNA) can be used to knock down the expression of *TCRP1*, and quantitative characterization of gene expression in *TCRP1* knock-down cells should provide new insights on regulatory pathways involved in the pharmacological action of *TCRP1*.

In this study, we examined genes that were differentially expressed in Tca/PYM and Tca/PYM-siTCRP1 cell lines by Human Toxicology and Drug Resistance Microarray (OHS-401) to obtain a global profile of drug-resistance genes regulated by *TCRP1*. Seven selected genes were analyzed by quantitative real-time PCR (Q-PCR) to verify the gene microarray results. We were intrigued by the finding that the most downregulated protein, metallothionein1X (MT1X), could bind with *TCRP1* in co-immunoprecpitation (co-IP) and GST-pull down assays. We next determined MT1X protein expression levels in primary oral squamous cell carcinoma and knocked down its expression in Tca/PYM cell by siRNA. Drug sensitivity studies were conducted to determine the influence of MT1X and *TCRP1* on the multidrug-resistant phenotype and apoptosis of OSCC cells. Finally, we sought to identify the pathway contributing to the anti-apoptosis effects of TCRP1.

## Results

### 1. *In vitro* Effects of TCRP1 Interfering RNA on Human OSCC

**Figure 1:**
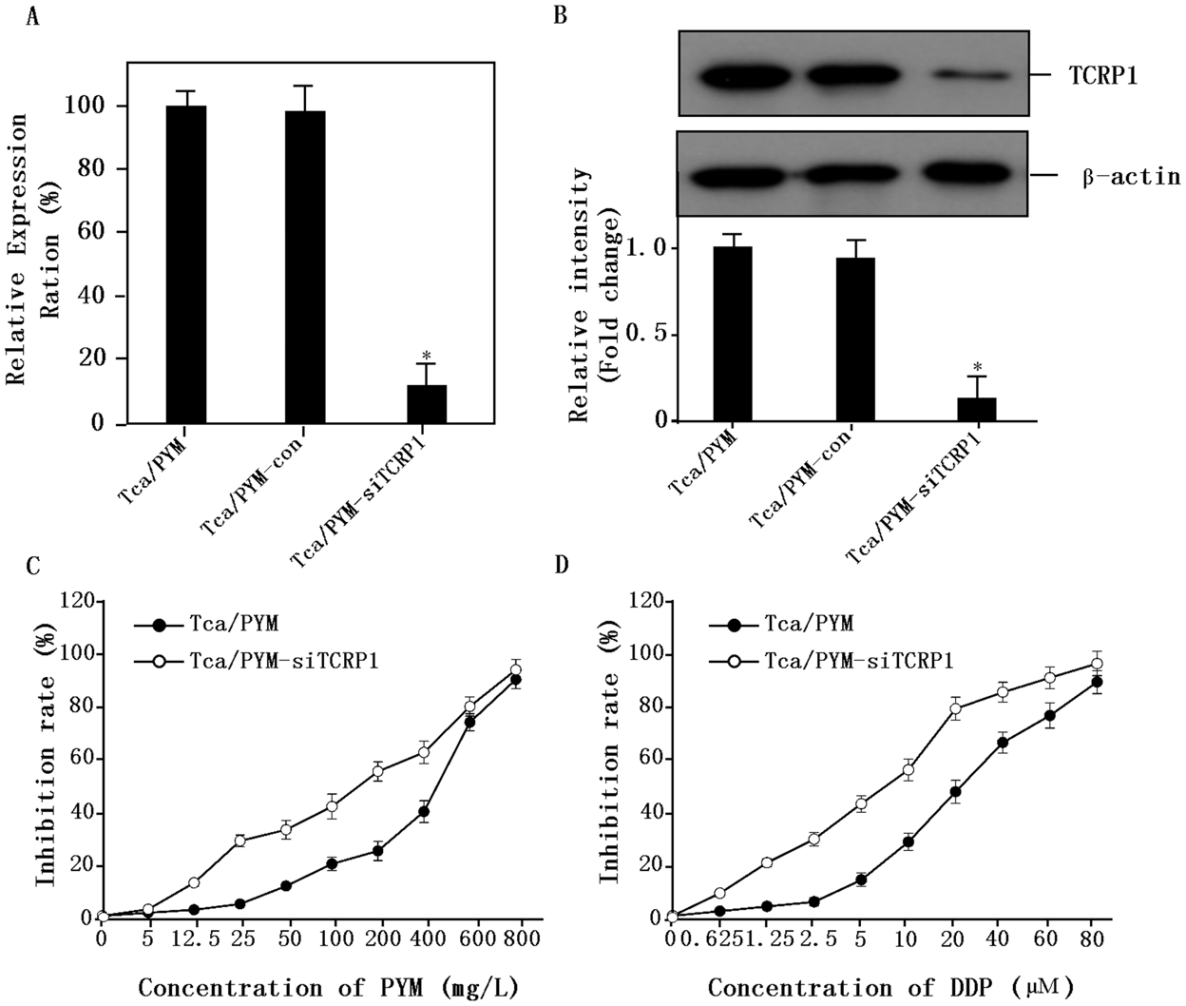
*In vitro* effects of TCRP1 interfering RNA on human OSCC. **A.** Real-time PCR analysis of TCRP1 mRNA expression. Treatment of Tca/PYM cells with TCRP1 interfering plasmid (pAU-siTCRP1) led to a significant decrease in TCRP1 mRNA levels. No reduction in expression was observed for untreated cells or cells treated with scrambled interfering plasmid. **B.** TCRP1 expression in Tca/PYM, Tca/PYM-Con, and Tca/PYM-siTCRP1 cells were examined by Western blot. Decreased expression of TCRP1 was observed after transfection with TCRP1 interfering plasmid in Tca/PYM cells. No reduction in expression was observed for untreated cells or cells treated with scrambled interfering plasmid. β-actin was used as the loading control. **C.** Responses of Tca/PYM and Tca/PYM-siTCRP1 cells to PYM. Tca8113/PYM cells were more resistant to PYM. **D.** Responses of Tca/PYM and Tca/PYM-siTCRP1 cells to DDP. Tca8113/PYM-siTCRP1 cells were more sensitive to DDP.

We first assessed the effects of knocking down the endogenous level of TCRP1 protein by pAU-siTCRP1 interfering plasmid transfected using Lipofectamine2000. Positive colonies were selected using G418 and the cell lines named Tca/PYM-Con and Tca/PYM-siTCRP1. Then, we examined TCRP1 expression using real-time PCR and Western blot analysis. Compared with no treatment or treatment with control plasmid, transfection with the pAU-siTCRP1 plasmid caused a marked decrease in *TCRP1* mRNA levels (Figure 1A). Similarly, TCRP1 protein levels decreased post-transfection, whereas no reduction was observed in untreated or control plasmid-treated cells (Figure 1B). These data indicated that our TCRP1-interfering plasmid could successfully diminish TCRP1 expression at the mRNA and protein levels.

To examine the role of TCRP1 in multidrug resistance, the cells were treated with different concentrations of PYM and DDP, and dose-response curves were plotted. As shown in Figure 1C and 1D, dose-dependent anti-proliferative activities were observed in Tca/PYM and Tca/PYM-siTCRP1 cell lines. However, the sensitivity of Tca/PYM-siTCRP1 to DDP was 3.29-fold higher than that of the parental Tca/PYM cells, as measured by the IC_50_ values for DDP over 72 h treatments. By contrast, the IC_50_ value of Tca/PYM-siTCRP1 to PYM was 2.57-fold higher. These data show that treatment with the TCRP1 interfering plasmid complex inhibits the proliferation of OSCC cells *in vitro*.

### 2. Differentially Expressed Genes in TCRP1 Knockdown Tca/PYM Cells

**Figure 2:**
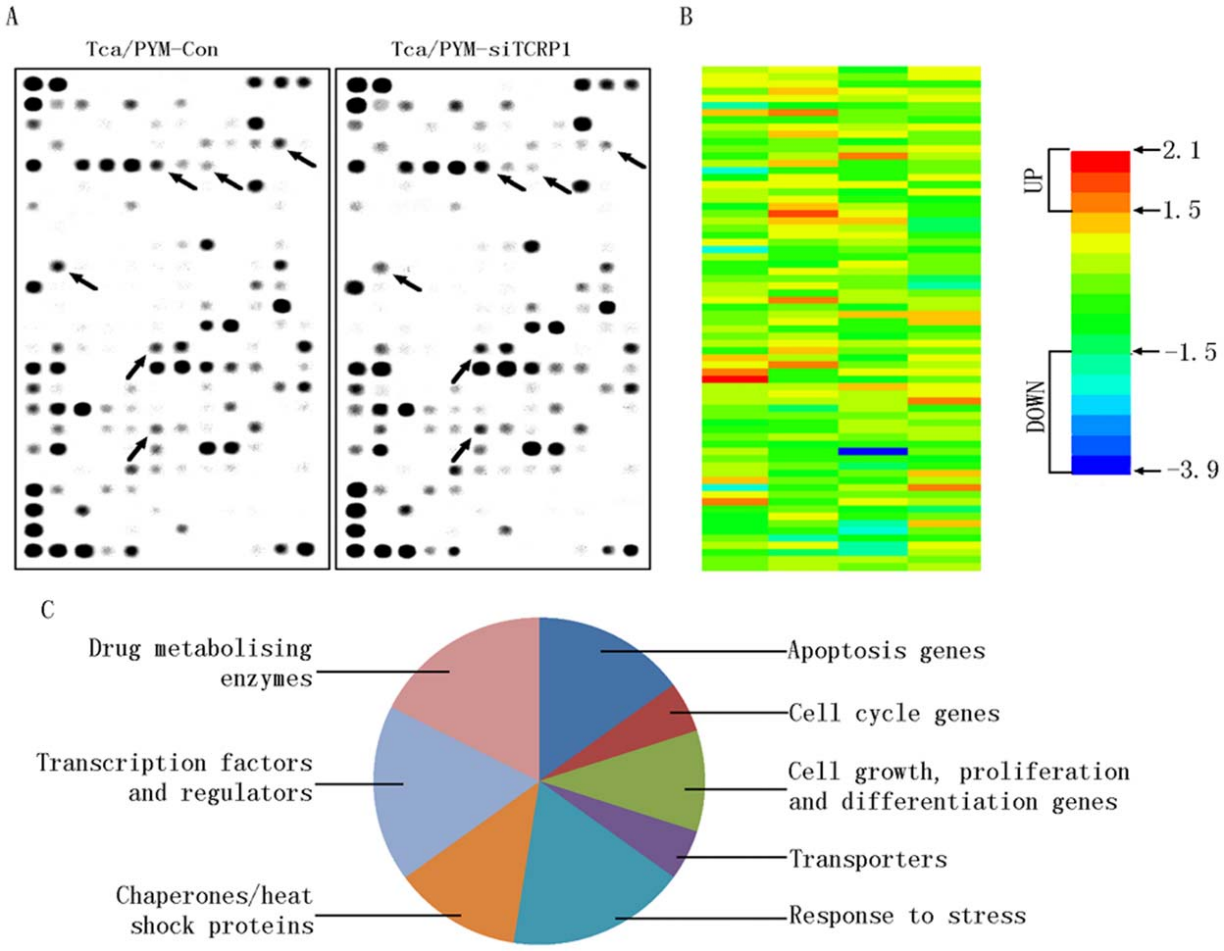
Identification of genes potentially involved in TCRP1-mediated multidrug-resistance phenotype. **A.** Differentially expressed genes in the Tca/PYM-Con (left) and Tca/PYM-siTCRP1 (right) cells were analyzed by Human Toxicology and Drug Resistance Microarray (OHS-401). This microarray included 263 key genes critical in drug metabolism and resistance. **B.** A heat map generated from the microarray shows gene expression as Tca/PYM-siTCRP1 over Tca/PYM-Con cells for the genes whose expressions had increased or decreased by more than 1.5-fold in TCRP1 knockdown cells. **C.** GO analysis of functional gene grouping of the differentially expressed genes involved in TCRP1-associated multidrug-resistance phenotype.

To characterize genes that contribute to TCRP1-associated multidrug-resistance, we used Human Toxicology and Drug Resistance Microarray to compare gene expression profiles between Tca/PYM and TCRP1 knockdown cell lines (Tca/PYM-siTCRP1) (Figure 2A). We chose genes that showed more than 1.5-fold change in expression in TCRP1 knockdown cells compared with the parent cells (Figure 2B). We found that expression of 30 genes was changed in the TCRP1 interfering cells compared with the original population. Among them, nine genes were upregulated with changes ranging from 1.519- to 2.076-fold (Table 1), whereas 21 genes were downregulated with changes ranging from -1.513- to -3.889-fold (Table 2). The *CCT4* gene, which showed strongest upregulation, increased by 2.076-fold, whereas the *MT1X* gene, which showed the strongest downregulation, decreased by 3.889-fold. These genes were grouped into the following eight categories according to their functions as described by GO analysis (Figure 2C): 1) apoptosis, 2) cell cycle, 3) cell growth, proliferation, and differentiation, 4) transporters, 5) response to stress, 6) chaperones/heat shock proteins, 7) transcription factors and regulators, and 8) drug metabolizing enzymes. This diversity is consistent with the existence of numerous regulatory mechanisms associated with *TCRP1* in response to drug resistance. Many of the upregulated genes are associated with chaperones/heat shock proteins (*CCT3*, *CCT4*, *DNAJA2*, and *DNAJC4*) and drug metabolizing enzymes (*CYP1A1*, *CHST4*, and *CYP3A5*). Among the downregulated genes, many are associated with apoptosis (Akt1, *NFκB1*, *NFκB2*, GADD45B, *NFκBIA*, *NFκBIB*, and TRADD). Housekeeping genes were not found to be differentially expressed.

**Table 1:** Genes with upregulated expression in Tca/PYM-siTCRP1 cells.

No	Symbol	GenBank ID	Ratio	Function^a^	Description
1	CCT4	NM_006430	2.076	2,6	Chaperonin containing TCP1, subunit 4(delta)
2	CYP3A5	NM_000777	1.878	8	Cytochrome P450, family3, subfamily A, polypeptide 5
3	DNAJA2	NM_005880	1.631	6	DnaJ (Hsp40) homolog, subfamily A, member 2
4	CCT3	NM_005998	1.629	6	Chaperonin containing TCP1, subunit 3 (gamma)
5	TP53	NM_00546	1.623	1,2,7	Tumor protein p53
6	HIF1A	NM_001530	1.587	4	Hypoxia-inducible factor 1, alpha subunit (basic helix-loop-helix transcription factor)
7	CHST4	NM_005769	1.580	8	Carbohydrate (N-acetylglucosamine 6-O) sulfotransferase 4
8	CYP1A1	NM_000499	1.549	8	Cytochrome P450, family 1, subfamily A, polypeptide 1
9	DNAJC4	NM_005528	1.519	6	DnaJ (Hsp40) homolog, subfamily C,member 4

a Function: 1. Apoptosis genes; 2. Cell cycle genes; 3. Cell growth, proliferation and differentiation genes; 4. Transporters; 5. Response to stress; 6. Chaperones/heat shock proteins; 7. Transcription factors and regulators; 8. Drug metabolizing enzymes.

**Table 2:** Genes with downregulated expression in Tca/PYM-siTCRP1 cells.

No	Symbol	GenBank ID	Ratio	Function^a^	Description
1	MT1X	NM_005952	−3.889	5	Metallothionein 1X
2	CHST2	NM_004267	−2.318	8	Carbohydrate sulfotransferase 2
3	BCR	NM_004327	−2.197	3	Breakpoint cluster region
4	AKT1	NM_005163	−2.142	1,5	V-akt murine thymoma viral oncogene homolog 1
5	NFκB2	NM_002502	−2.097	1,3,7	Nuclear factor of kappa light polypeptide gene enhancer in B-cells 2 (p49/p100)
6	ABCC3	NM_003786	−2.023	4	ATP-binding cassette, sub-family C (CFTR/MRP), member 3
7	NFκB1	NM_003998	−1.871	1,5,7	Nuclear factor of kappa light polypeptide gene enhancer in B-cells 1 (p105)
8	SULT1A1	NM_001055	−1.870	8	Sulfotransferase family, cytosolic, 1A
9	GADD45B	NM_015675	−1.868	1,5	Growth arrest and DNA-damage- inducible, beta
10	NFκBIB	NM_002503	−1.842	1,7	Nuclear factor of kappa light polypeptide gene enhancer in B-cells inhibitor, beta
11	NNMT	NM_006169	−1.819	8	Nicotinamide N-methyltransferase
12	GDF15	NM_004864	−1.778	3	Growth differentiation factor 15
13	ST13	NM_003932	−1.740	6	Suppression of tumorigenicity 13 (colon carcinoma) (Hsp70 interacting protein)
14	TRADD	NM_003789	−1.697	1	TNFRSF1A-associated via death domain
15	EGFR	NM_005228	−1.696	3	Epidermal growth factor receptor
16	RXRB	NM_021976	−1.662	7	Retinoid X receptor, beta
17	MVP	NM_017458	−1.656	5	Major vault protein
18	RXRA	NM_002957	−1.600	7	Retinoid X receptor, alpha
19	NFκBIA	NM_020529	−1.561	1,5	Nuclear factor of kappa light polypeptide gene enhancer in B-cells inhibitor, alpha
20	AP1S1	NM_001283	−1.519	4	Adaptor-related protein complex 1, sigma 1 subunit
21	CHST8	NM_022467	−1.513	8	Carbohydrate (N-acetylgalactosamine 4-0) sulfotransferase 8

a Function: 1. Apoptosis genes; 2. Cell cycle genes; 3. Cell growth, proliferation and differentiation genes; 4. Transporters; 5. Response to stress; 6. Chaperones/heat shock proteins; 7. Transcription factors and regulators; 8. Drug metabolizing enzymes.

### 3. Confirmation of Microarray Results by Real-time PCR and Western Blot

**Figure 3:**
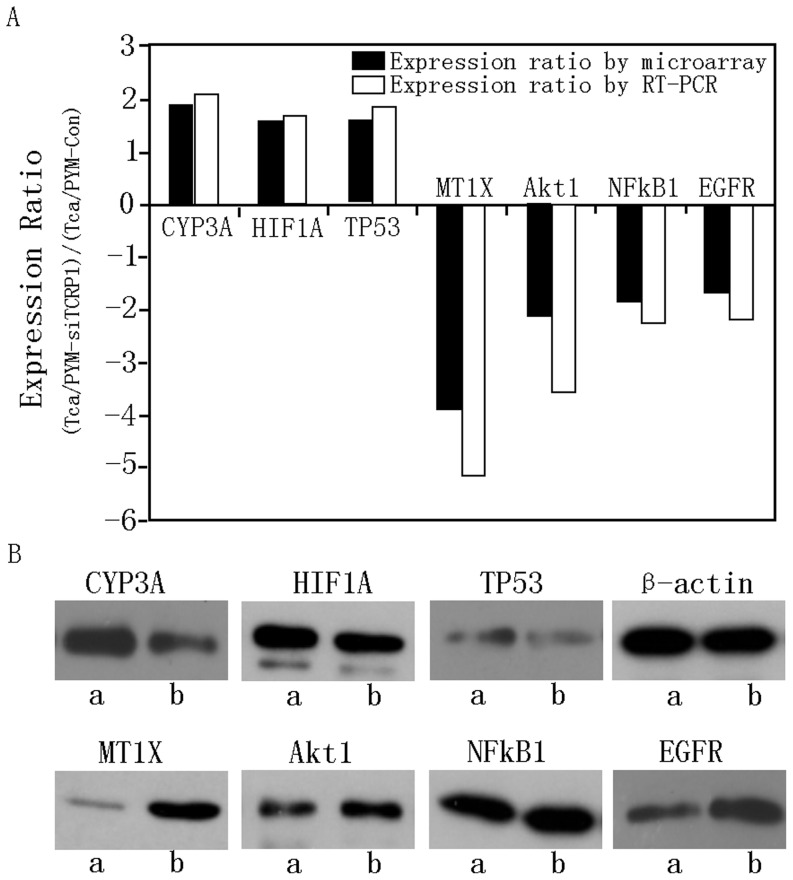
Validation of microarray results by real-time PCR and Western blot. **A.** Comparison of cDNA microarray expression ratios (Tca/PYM-Con expression over Tca/PYM-siTCRP1 expression) with those determined by real-time PCR for seven genes. The relative fold changes were calculated after normalizing against β-actin expression. Ratios of transcripts in Tca/PYM-Con and Tca/PYM-siTCRP1 cells were comparable to those obtained in the microarray analysis. Each column represents the results of three independent experiments. **B.** We further validated the expression of CYP3A5, HIF1A, TP53, MT1X, NFκB1, Akt1, and EGFR proteins in both cell lines, using β-actin as a loading control to investigate the correspondence between mRNA and protein levels for these different genes. Similar changes as those found in the microarray analysis were observed. Each figure represents three independent experiments (a: TcaPYM-siTCRP1 cells, b: Tca/PYM-Con cells).

To confirm the data generated from the microarray studies, we chose a subset of seven genes that were either highly upregulated (*CYP3A5*, *HIF1A*, and *TP53*) or downregulated (*MT1X*, *NFκB1*, *EGFR*, and *Akt1*) for validation by real-time PCR and Western blot. Our results confirmed our microarray analysis data: all seven differentially expressed genes were either upregulated (*CYP3A5*, *HIF1A*, and *TP53*) or downregulated (*MT1X*, *NFκB1*, *EGFR*, and *Akt1*). For example, the mean fold changes in upregulation as determined by microarray analysis and by real-time PCR were 3.889 and 5.253 for *MT1X*, and 1.878 and 2.137 for *CYP3A5*, respectively. Expression of MT1X, NFκB1, EGFR, Akt1, CYP3A5, HIF1A, and TP53 proteins was further tested by Western blot (Figure 3B). As shown in the results of three independent experiments in Figure 3, our Western blot analyses also confirmed the microarray results.

### 4. Akt and MT1X could Combine with TCRP1

**Figure 4:**
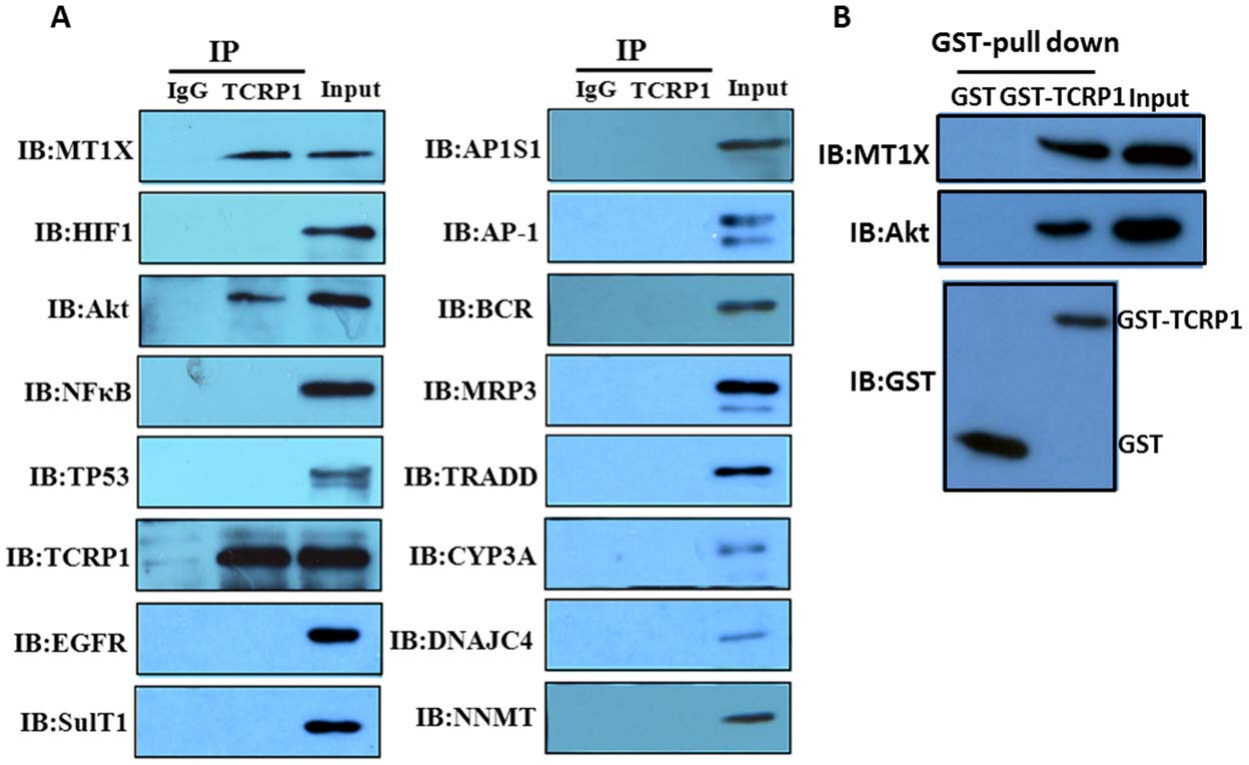
TCRP1 interacts with candidate proteins. **A.** Tca8113/PYM cell lysates were incubated with normal mouse IgG-conjugated agarose (control IgG) or anti-TCRP1 antibody-conjugated agarose (TCRP1). The immunoprecipitants and cell lysates (input) were electrophoresed and immunoblotted with the indicated antibodies or TCRP1. **B.** GST pull-down assay. GST alone or GST-tagged TCRP1 was incubated with Tca8113/PYM cell lysates. The precipitated proteins and the input proteins were detected by immunoblotting with antibodies to GST, Akt or MT1X.

Once differentially expressed genes were identified and confirmed by RT-PCR and Western blot, the significance of the changes in gene expression was further investigated. First, co-immunoprecipitation experiments were performed to determine whether any identified protein could bind with TCRP1. We chose 15 genes, including MT1X, NFκB, and Akt, as the target protein for co-immunoprecipitation (Figure 4A). Our results showed that MT1X and Akt could bind with TCRP1. We also carried out a pull-down assay using the recombinant protein to confirm this finding. TCRP1 was purified from bacteria as a GST fusion protein [7]. GST-tagged TCRP1 or GST alone was incubated with cell lysates. Pull-down analysis revealed that both MT1X and Akt formed a complex with GST-TCRP1 *in vitro* but not with GST alone (Figure 4B). These results show that MT1X and Akt can physically bind to TCRP1, suggesting that they might play important roles in TCRP1-associated drug resistance. It is noteworthy that MT1X was the most downregulated protein in Tca/PYM-siTCRP1 cells. Although many studies have evaluated the role of metallothionein (MT) as a heavy-metal detoxifier in the resistance of tumors to anti-cancer drugs, and high levels as well as induction of MT have been found to coincide with protection against damage by heavy metals, data on the involvement of MT1X in acquired resistance to DDP is far from consistent. Therefore, we chose to further study the role of MT1X in TCRP1-associated drug resistance.

### 5. Expression of TCRP1 and MT1X in Primary Oral Squamous Cell Carcinoma

In our previous work, we had classified 60 clinical samples into cisplatin-sensitive and cisplatin-resistant groups on the basis of growth inhibition, and had found up-regulation of TCRP1 in the resistant group and opposite changes in the sensitive group [7]. In the current study, immunostaining results were ranked according to the extent of TCRP1 immunoreactivity: ‘−’ (0–5%), ‘+’(5%–20%), ‘++’ (20%–50%), and ‘+++’ (>50%). The Mann–Whitney U test was carried out to identify whether there is any correlation between MT1X expression levels with drug resistance patterns. As shown in table 3, MT1X expression level in the 30 cisplatin-sensitive cases were as follows: 36.67% were graded as ‘−’, 40.00% as ‘+’, 13.33% as ‘++’, and 10.00% as ‘+++’. Of the 30 cisplatin-resistant cases, 13.33% were graded as ‘−’, 10.00% as ‘+’, 43.33% as ‘++’, and 33.33% as ‘+++’. In other words, the expression level of MT1X in cisplatin-sensitive and resistant groups have significant difference with Mann–Whitney U test P value less than 0.001. These results were consistent with our previously research about TCRP1 (*P* = 0.005) [7]. Meanwhile, Spearman’s correlation coefficient was used to analyze whether the relationships between the expression patterns of TCRP1 and MT1X in primary tumors correlate with their drug resistance patterns (Tab 3). The results shown the expression of TCRP1 and MT1X in cisplatin-sensitive groups were not statistically significant (r = 0.886, *P* = 0.114), but cisplatin-resistant groups were statistically significant (r = 0.990, *P* = 0.01). These results indicated that it has a positive correlation between TCRP1 and MT1X expression in the cisplatin-resistant clinical samples.

**Table 3:** Statistically significant molecular and immunohistochemical parameters.

Group	Total Case	TCRP1 IHC Scores				MT1X IHC Scores			
		−	+	++	+++	−	+	++	+++
**Resistant**	**30**	**5**	**4**	**11**	**10**	**4**	**3**	**13**	**10**
**Sensitive**	**30**	**11**	**10**	**7**	**2**	**11**	**12**	**4**	**3**
		a **TCRP1 ** ***P*** ** = 0.005**				a **MT1X ** ***P*** ** = 0.000**			
bResistent group: r = 0.990,*P* = 0.01 Sensitive group: r = 0.886,*P* = 0.114									

Clinical samples from primary oral squamous cell carcinoma were classified into cisplatin-resistant and cisplatin-sensitive groups on the basis of MTS assays as described in Materials and Methods.

a Mann–Whitney U test was used to compare the differences in TCRP1 and MT1X with varying degrees of IHC Scores (−, +, ++, +++) between the resistant and sensitive groups. *P*-values for statistical significance are indicated.

b Spearman’s correlation coefficient was used to analyze whether the relationships between expression of TCRP1 and MT1X in primary tumors correlate with their drug resistance patterns. *P*<0.05 was considered statistically significant.

### 6. Reduction in MT1X Activity Sensitizes Cells to DDP

**Figure 5:**
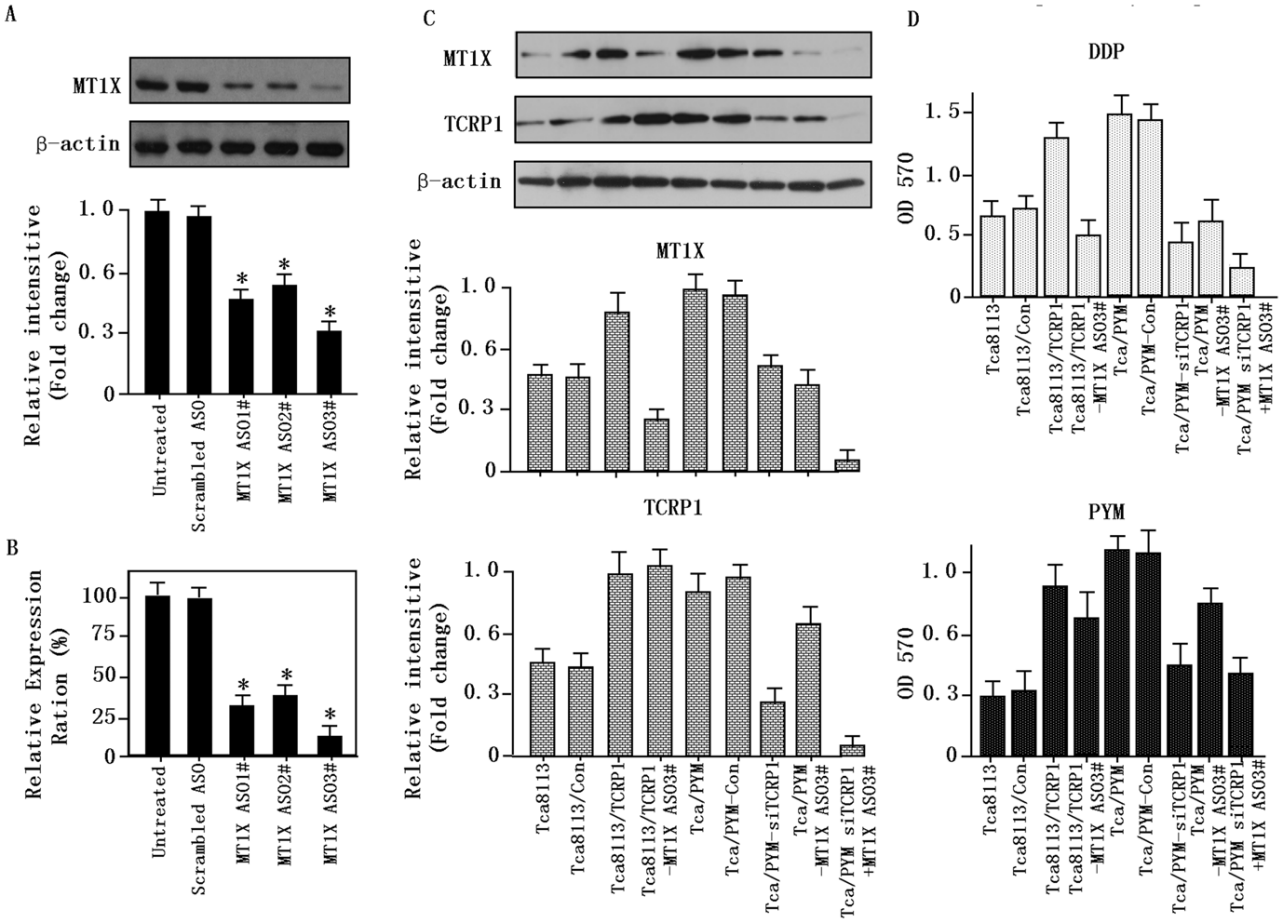
Reduction in MT1X activity sensitizes cells to DDP *in vitro*. **A.** MT1X expression in Tca/PYM cells was examined by Western blot. Decrease in expression of MT1X was observed after transfection with MT1X interfering RNA in Tca/PYM cells. No reduction in expression was observed in cells that were treated with scrambled interfering RNA or in untreated cells. β-actin was used as the loading control. **B.** MT1X expression in Tca/PYM cells was examined by Real time-PCR. **C.** MT1X and TCRP1 expression in genetically engineered Tca8113 cells were determined by Western blot. **D.** Responses of different cells to DDP and PYM were determined by MTS. Each point represents the mean of data from three independent experiments.

We next determined whether MT1X is required for TCRP1-mediated DDP resistance. Tca/PYM cell lines were transfected with MT1X siRNA or scrambled siRNA (100 nmol/L) for 48 h. After treatment, we examined MT1X expression using real-time PCR and Western blot analysis. Compared with untreated or scrambled siRNA-treated cells, all three MT1X siRNAs caused marked decrease in MT1X mRNA and protein levels (Fig. 5A and 5B). MT1X ASO3# exhibited the strongest knockdown effects among the three siRNAs so it was used in all subsequent experiments.

We also evaluated MT1X expression level in genetically engineered Tca8113 cells. MT1X expression was found to correlate with TCRP1 expression, for example, the Tca8113/TCRP1 and the Tca/PYM cell lines, which have high expression of TCRP1, showed high expression of MT1X as well (Fig. 5C).

Finally, we examined whether MT1X could inhibit DDP activities in the different cell lines to characterize the role of MT1X in DDP resistance. MTS results showed that knockdown of MT1X expression in Tca/PYM cells increased their sensitivity to DDP, but had no obvious effects on their response to PYM (Fig. 5D). These findings suggest a correlation between expression of TCRP1 and MT1X with DDP resistance in OSCC cells.

### 7. MT1X Knockdown Affects Cellular Apoptosis and Soft Agar Colony Formation

**Figure 6:**
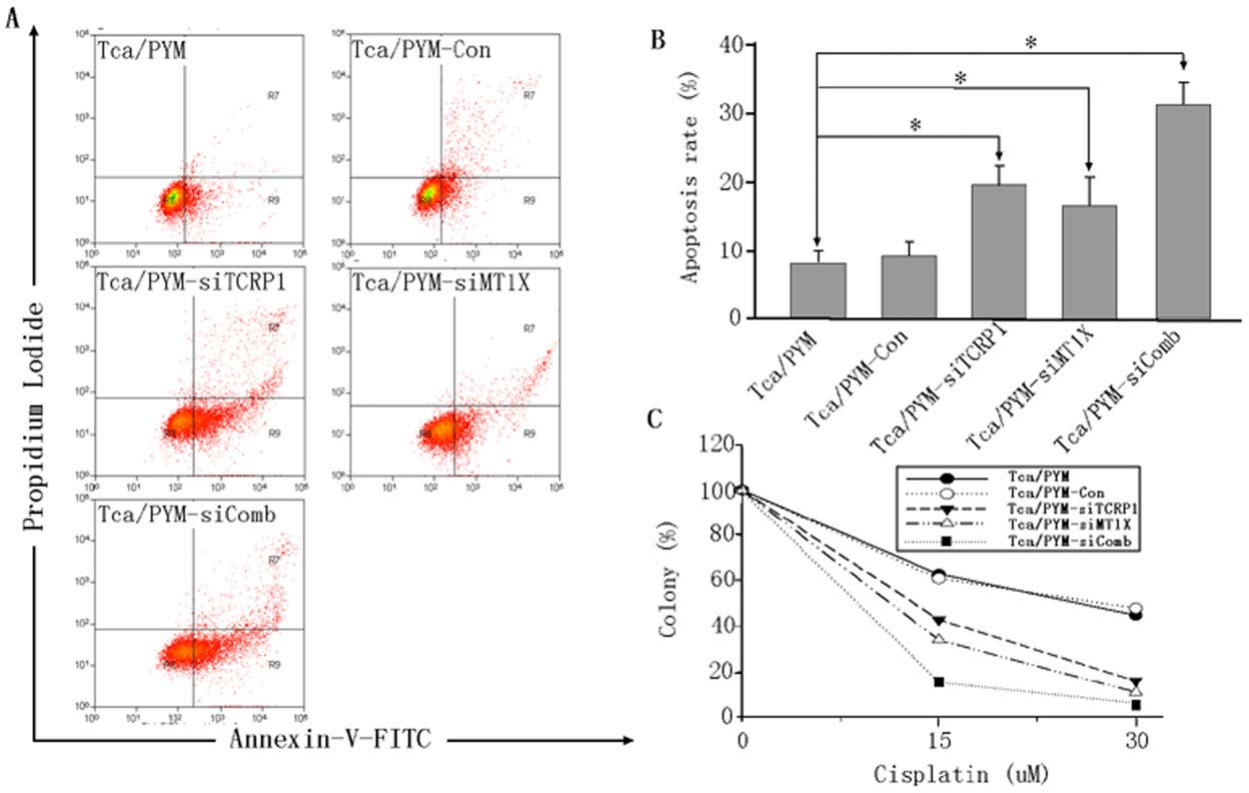
MT1X knockdown affects cellular apoptosis and soft agar colony formation. **A.** Cells were treated with DDP for 48 h as described in Materials and Methods. Apoptotic cells were determined by flow cytometry after PI and Annexin-V-FITC double staining (Tca/PYM-siComb = Tca/PYM-siTCRP1 + MT1X ASO3#). **B.** Quantitative analysis of apoptosis data. Data represents mean ± S.D. of three independent experiments. *P<0.05 versus untreated control. **C.** TCRP1 knockdown affects anchorage-independent growth of Tca/PYM, Tca/PYM-Con, and Tca/PYM-siTCRP1 cells. No. of colonies (>50 cells) per 35 mm^2^ dish relative to those of untreated control cells were calculated. The assay was done in biological triplicates (soft agar plating in duplicate for each biological replicate; n = 3). *P<0.05 versus untreated control.

A major mechanism of DDP-induced tumor killing is the induction of apoptosis [13]. Our gene function category analysis also identified Tca/PYM cell line to have enrichment of apoptosis-associated genes (Fig. 2C). In order to examine whether MT1X is involved in apoptosis, we examined the extent of apoptosis in Tca/PYM cells treated with 20 µM DDP for 24 h by Annexin-V FITC and PI double staining. Significant increases in the percentage of apoptotic cells from 8.04% to 15.47% and 20.20% were observed when the cells were treated with siMT1X and siTCRP1 RNA, respectively. When we treated the Tca/PYM cell combination with siMT1X and siTCRP1, the percentage of apoptotic cells increased to 37.48% (Fig. 6A,B). These results indicate that *TCRP1* is an anti-apoptotic gene and can block DDP-induced apoptosis in OSCC tumor cells. To evaluate whether MT1X could confer resistance to Tca/PYM cell lines, we performed colony-forming assays in Tca/PYM cells transfected with siRNAs against MT1x for 48 h. As shown in Figure 6C, cells in which MT1X was knocked down tended to form fewer colonies than control cells after cisplatin exposure for 24 and 48 h.

### 8. Activation of NFκB Contributes to the Anti-apoptotic Effect of TCRP1

**Figure 7:**
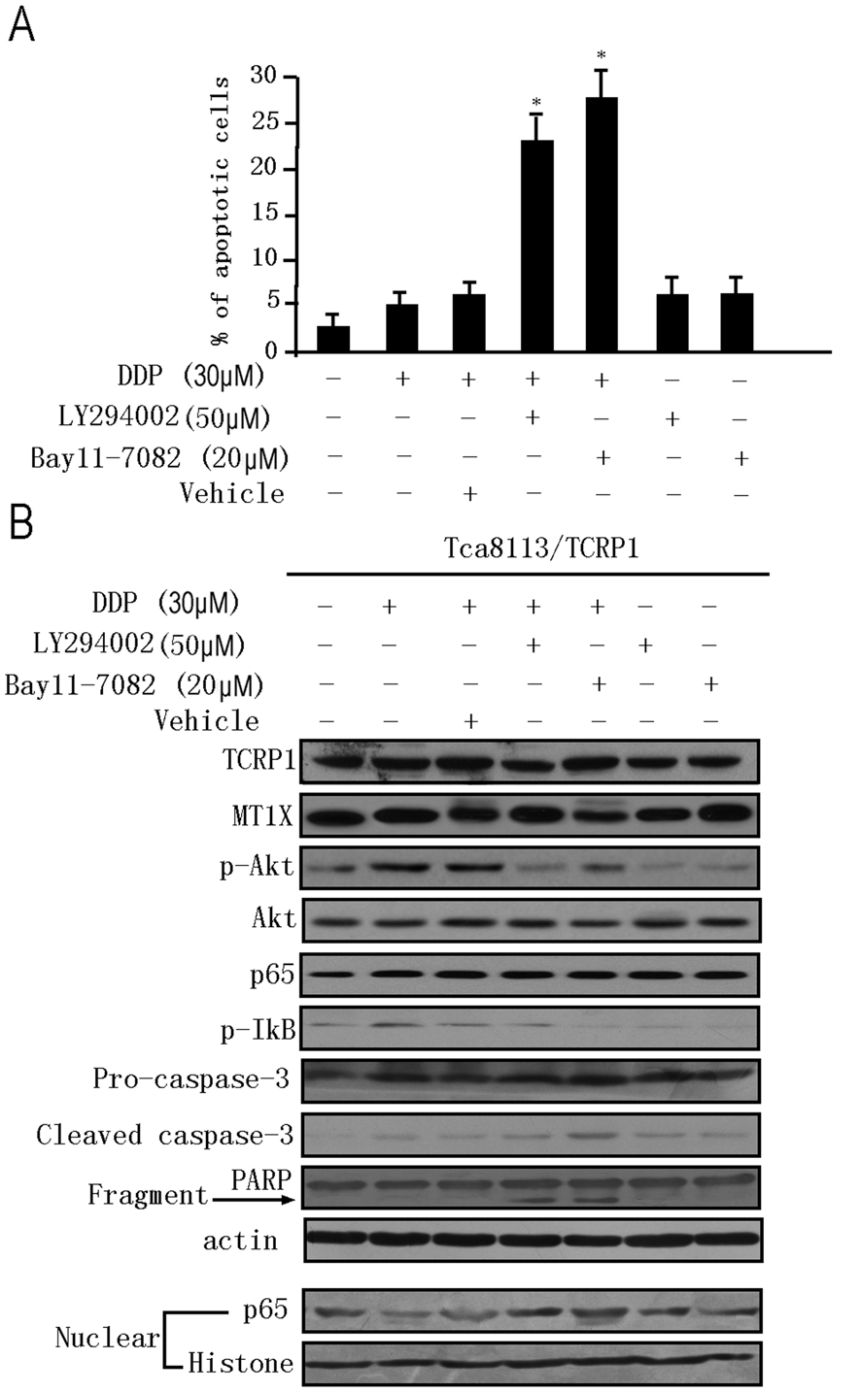
TCRP1 regulates the PI3K/Akt/NFκB pathway and protects oral squamous cells from cisplatin induced apoptosis. TCRP1-over-expressing cells (Tca/TCRP1) were treated with 30 µM DDP in the presence or absence of specific inhibitors of PI3K (LY2294002 [50 µM]), NFκB (Bay11-7082 [20 µM]) or vehicle (DMSO) and cultured for an additional 24 h. **A.** The degree of apoptosis was determined by flow cytometry as described in Figure 6. Significantly higher percentages of apoptotic cells were found in the presence of both inhibitors (*P<0.05, compared with control). **B.** Western blot analyses showing the effect of TCRP1 on apoptosis associated proteins in Tca/TCRP1 cells. Nuclear histone and β-actin were included as loading controls. All experiments were repeated at least three times, and representative results are shown.

Our microarray results showed that many NFκB family proteins (NFκB1, NFκB2, NFκBIA, and NFκBIB) were down-regulated when TCRP1 expression was knocked down. Notably, Akt and some types of MT are known to prevent activation of the NFκB signaling pathway which plays a significant role in preventing cells from undergoing apoptosis [14], [15], [16]. These observations led us to hypothesize that Akt/NFκB signaling pathway may contribute to the anti-apoptotic effect of TCRP1. To confirm this hypothesis, we treated TCRP1-over-expressing cells (Tca8113/TCRP1) with 30 µM of cisplatin in the presence of specific inhibitors of PI3K (LY2294002 [50 µM]) or NFκB (Bay11-7082 [20 µM]), or of vehicle (DMSO) and cultured for 24 hours. As shown in Figure 7A, treatment with both inhibitors elicited significant apoptotic response in Tca8113/TCRP1 cells compared with the vehicle control (2.3% versus 23.4% and 27.7% apoptotic cells in LY2294002 and Bay11-7082, respectively). These results indicated that activation of NFκB may play a significant role in protecting Tca8113/TCRP1 cells from apoptosis.

To provide further proof that activation of Akt/NFκB mediates the anti-apoptotic effects of TCRP1, we investigated the expression of Akt/NFκB pathway proteins by Western blot. Since NFκB translocates to the nucleus upon activation, we studied the levels of the most abundant subunit of NFκB, p65, in nuclear extracts and whole lysates of TCRP1-over-expressing cells. Our Western blot results showed that inhibition of the PI3K/Akt/NFκB signaling pathway using specific inhibitors led to increased expression of apoptotic markers, such as cleaved caspase-3 and PARP (Figure 7B). Additionally, when Tca8113/TCRP1 cells were treated with DDP, regardless of whether the PI3K/Akt or the NFκB part of the PI3K/Akt/NFκB pathway was inhibited, increased apoptosis was observed. Meanwhile, treatment with inhibitors of PI3K (LY2294002) or NFκB (Bay11-7082) did not affect TCRP1 and MT1X expression, suggesting that TCRP1 and MT1X act upstream of Akt/NFκB. Taken together, these results illustrate that TCRP1 functions as an upstream regulator of the NFκB signaling pathway, and the PI3K/Akt/NFκB pathway plays an important role in mediating the anti-apoptotic effect of TCRP1.

## Discussion

The aim of the present work was to characterize in more detail the role of the TCRP1 pathway in the development of DDP resistance. We applied high-throughput functional gene microarray analysis to assess the role of TCRP1 associated signaling pathways in OSCC cell lines. Gene expression profiles of human PYM-resistant cell lines were compared with those in which TCRP1 was knocked down by siRNA. We found that the TCRP1 knockdown cell line, Tca/PYM-siTCRP1, had a genome-wide expression profile that differed from that of the Tca/PYM cell line. Using the function gene microarray (OHS-401), we identified 30 genes that had significantly different expression levels between Tca/PYM and Tca/PYM-siTCRP1 cells. Considering that Tca/PYM-siTCRP1 and Tca/PYM cells have identical genetic backgrounds, we presume that differences in expression of these genes are related to TCRP1 expression, and that these differences might shed light on the mechanism of Tca/PYM resistance to DDP.

Of the 30 candidate genes, 9 showed higher expression and 21 showed lower expression in TCRP1 knockout cells compared with Tca/PYM. Gene function category analysis revealed that the Tca/PYM cell line, which has high TCRP1 expression, has an enrichment of apoptosis-associated genes. Induction of apoptosis has been reported as an important factor in determining response to chemotherapy [13], [17], [18]. Notably, some of the genes with altered expression in TCRP1 knockdown cell lines were apoptosis-related genes such as *AKT*, *NFκB*, *BCR*, *TRADD*, and *CCT4.* All of them had significantly lower expression in Tca/PYM-siTCRP1 cells than in Tca/PYM cells. *MT1X* was downregulated the most after TCRP1 interference. Mammalian metallothionein (MT) is a small protein of 61–62 amino acids that contains 20 cysteine residues. It has been suggested to have a role in the detoxification of heavy metal ions. Several lines of evidence suggest that MT is chemotherapy-inducible, and its expression constitutes a protective mechanism that prevents cells apoptosis induced by DDP and doxorubicin [19]. Both cis-DDP and trans-DDP have been shown to chelate with MT [20], [21], [22]. Although high levels of MT have been found to coincide with protection against damage by heavy metals, the involvement of MT in acquired resistance to DDP is less clear [23]. In our previous study, we established a human MT2A recombinant with soluble high-yield expression and demonstrated its hydroxyl radical-scavenging ability and significant protective role against DNA damage caused by UVC radiation [24]. In the present study, we found that downregulating the expression of *MT1X*, which is one isoform of MT, by siRNA can sensitize cells to DDP, in part via promotion of cell apoptosis and inhibition of clone formation (Figure 6).

Another important apoptosis-related gene associated with TCRP1 is the NF-κB family protein. *NF-κB* includes various dimeric complexes of members of the Rel protein family, which comprises of Rel (c- Rel), Rel A (p65), Rel B, NF-κB1 (p50 and its precursor p105), and NF-κB2 (p52 and its precursor p100). *NF-κB* regulates the expression of a variety of proteins that inhibit apoptosis and promote cell survival/proliferation [25], [26]. *Akt* is another major contributor to chemoresistance in human cancer [27]. Several studies have demonstrated that the *NF-κB* and *Akt* signaling pathways can converge [28], given that IκBα kinase, involved in NF-κB activation, is a substrate of *Akt.* Therefore, activation of Akt can stimulate NF-κB activity. Our previous studies showed that TCRP1 plays a significant role in mediating OSCC radio-resistance by upregulating the activity and levels of *Akt* in addition to elevating the level of NF-κB [8]. In the current study, we found that expression of Akt and several NF-κB members (NFκB1, NFκB2, NFκBIA, NFκBIB) was lower in Tca/PYM-siTCRP1 cells than in Tca/PYM cells. Further investigation indicated that TCRP1 induces activation of the PI3K/Akt/NF-κB signaling pathway, and this may be one of the mechanisms responsible for protecting Tca8113/TCRP1 cells from cisplatin-induced apoptosis. Future studies should address how TCRP1 activates the PI3K/Akt/NF-κB signaling pathway, and how AKT, NF-κB family, MT1X and TCRP1 are related functionally.

Our microarray results also indicated that increased drug detoxification is an important way for *TCRP1* associated with DDP resistance in OSCC. For example, many studies have evaluated the role of MT as a heavy-metal detoxifier in the resistance of tumors to anti-cancer drugs [29]. Human cytosolic sulfotransferase (SULT) enzymes catalyze the sulfate conjugation of many drugs [30]. Our results indicate that *TCRP1* could increase DDP detoxification by upregulating MT1X and SULT1A1. Cytochrome P450 3A subfamily are the most abundantly expressed CYP enzymes in the human liver. In adults, CYP3A4 and CYP3A5 are predominant among the four known isoforms (CYP3A4, CYP3A5, CYP3A7, and CYP3A43) [31]. The metabolism of 37% of all currently approved cytostatic and/or cytotoxic anticancer agents is known to be mediated, at least partially, by the CYP3A isoforms [31], [32]. Interestingly, it has been shown that *TCRP1* can promote CYP3A4 and CYP1A1 expression. The mechanism underlying these observations remains to be determined.

Several membrane transporters are known to confer multidrug resistance [33]. They do so by mediating energy-dependent reduced influx or increased efflux of drugs [34]. In our work, two membrane proteins were found to be downregulated in Tca/PYM-siTCRP1 cells. One is AP1S1, and the other is ABCC3, which encodes multidrug resistance-associated protein 3 and shows significant positive correlation with TCRP1-associated DDP resistance. This finding is consistent with a previous study that had indicated that overexpression of ABCC3 results in increased resistance to DDP [33], [35], [36]. Since we had confirmed in our previous report that TCRP1 is not a membrane protein [7], we speculate that TCRP1 is involved in DDP transport from the cytoplasm to the outside of cells via other membrane proteins such as AP1S1 and ABCC3.

**Figure 8:**
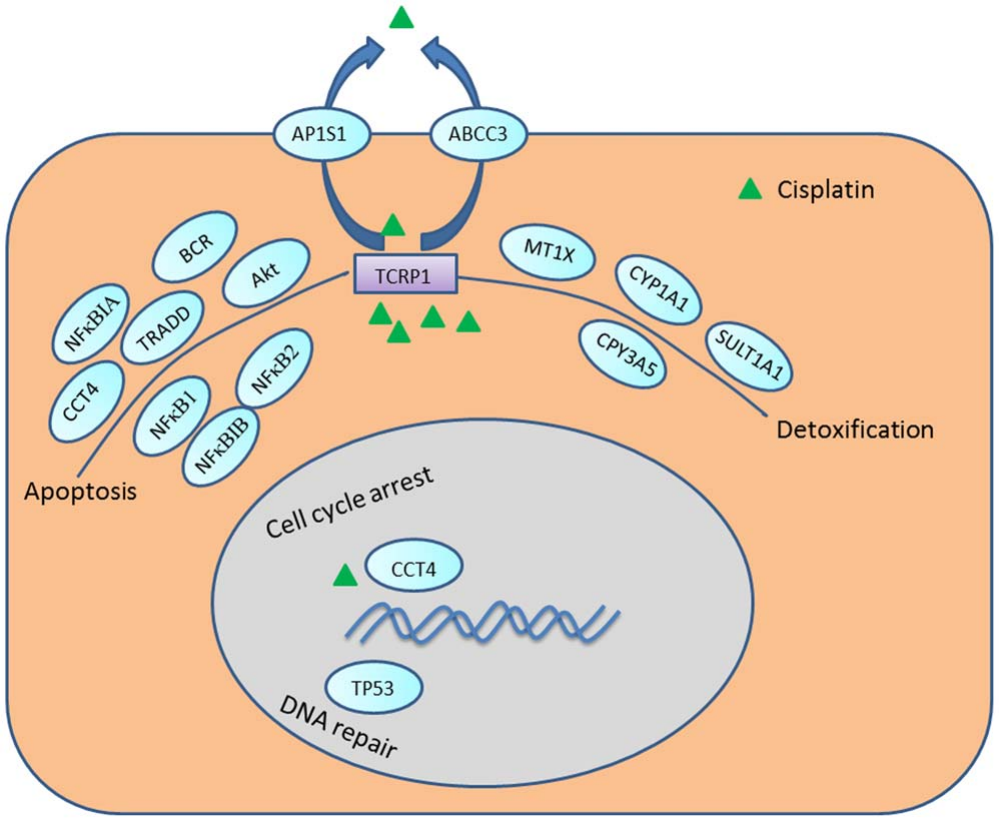
Representation of the candidate genes involved in the metabolism of DDP based on our microarray results.

In summary, our results indicate that *TCRP1* may participate in DDP resistance in OSCC by several mechanisms, (Figure 8) including 1) reduction in cellular accumulation, 2) inhibition of apoptosis, 3) reduction in the number of DNA intraqstrand crosslinks, and 4) enhancement of DNA repair, among others. Knocking down TCRP1 and MT1X by siRNA could sensitize cells to cisplatin through increased cancer cell apoptosis and inhibition of cell proliferation. This may be caused at least in part by TCRP1 induced activation of the PI3K/Akt/NF-κB signaling pathway. Our data suggest that inhibitors of both MT1X and TCRP1 may have potential therapeutic application in inducing apoptosis in OSCC. Although further *in vivo* validations for the identified genes are required, our findings may help in the development of better cancer chemotherapy strategies. Future work to examine the effectiveness of *TCRP1* knockdown on OSCC in preclinical animal models will help determine whether this approach represents a potential avenue for clinical trials.

## Materials and Methods

### Reagents

Chemicals and reagents for cell growth were purchased from Sigma-Aldrich (St. Louis, MO, USA). Promega Corporation (Madison, Wisconsin, USA) supplied reagents for RNA isolation, reverse transcription, PCR, restriction enzymes digestion, and ligation. Bio-Asia Biotechnology Co., Ltd. (Shanghai, China) synthesized primers for amplification. Antibodies against MT1X (sc-12807), Sult1 (sc-130883), CYP3A (sc-53616), MRP3 (sc-59613), AP-1(sc69446), TRADD (sc-7868), BCR (sc-886), DNAJC4 (sc-242591), NNMT (sc-48614) were purchased from Santa Cruz Biotechnology. The antibody against EGFR (352901) was purchased from Bio-Legend. TCRP1 polyclonal antibody was prepared by us as described previously [7]. The Bradford protein assay kit was purchased from Bio-Rad (California, USA).

### Cell Lines and Cell Culture

Human OSCC cell line Tca8113 was obtained from China Center for Type Culture Collection (Wuhan, China). PYM-induced MDR cell line Tca8113/PYM [5] and TCRP1 overexpression cell line Tca8113/TCRP1 [6] were previously established in our laboratory. The cells were cultured in RPMI1640 medium (Gibco, USA) supplemented with 10% heat-inactivated fetal bovine serum (Hyclone Corp., USA), 100 mg/ml streptomycin and 100 U/ml penicillin (Invitrogen) at 37°C with 5% CO_2_ in a humidified environment. Tca8113/PYM cells were routinely cultured under the same conditions with additional 100 ng/ml PYM (Harbin Bolai Pharmaceutical Co., China). Tca8113/PYM cells were maintained in PYM-free medium for at least 2 wk before the experiments.

### Construction of shRNAi Silencing Plasmids

We used the siRNA designing tool available on the website of Ambion Inc. (Austin, TX) to design the siRNA-encoding minigenes for TCRP1. Two complementary oligonucleotide DNA sequences (5′-TCGACAACAGCATTCCCTCTGCTATCTTCAAGAGAGATAGCAGAGGGAATGCTGTTTTTTT-3′ and 5′-CTAGAAAAAAACAGCATTCCTGCTATCTCTCTTGAAGATAGCAGAGGGAATGCTGTTG-3′) were synthesized with Sal I- and Xba I-compatible overhangs that facilitated their ligation into the expression vector pAU6+27 (gift from Professor David Engelke, University of Michigan). The resulting plasmid was named pAU-siTCRP1. The control plasmid encoding a nonsense minigene with no homology to any known sequence in the human genome was cloned using the same strategy (5′-TCGACAGCTTCATAAGGCGCATGCTTCAAGAGAGCATGCGCCTTATGAAGCTTTTTT-3′ and 5′-CTAGAAAAAAGCTTCATAAGGCGCATGCTCTCTTGAAGCATGCGCCTTATGAAGCTG-3′). Tca8113/PYM cell lines were transfected with pAU-siTCRP1 and the corresponding control plasmid using Lipofectamine 2000 according to the manufacturer’s instructions (Invitrogen). Clones were generated by stable transfection. Stable clones were generated by transfection of expression vectors and/or siRNA plasmids and were selected by G418. The resulting cell lines were named Tca81113/PYM-siTCRP1 and Tca8113/PYM-Con.

For siRNA against MT1X, three sequences of MT1X ASOs corresponding to different sites of human MT1X were selected, and a scrambled oligonucleotide was used as control. The transfection was performed with Lipofectamine 2000 (Invitrogen) in accordance with the manufacturer’s instructions. MT1X expression was determined by real-time PCR and Western blot 2 days post-transfection. Cell viability was examined by MTS assay after DDP or PYM treatment.

### RNA Preparation and Affymetrix Genechip Hybridization

Total RNA was extracted using the Trizol reagent (Invitrogen Inc., Carlsbad, CA) according to the manufacturer’s instructions. Genes expressed in the Tca8113/PYM-Con and Tca8113/PYM-siTCRP1 cell lines were analyzed on an Oligo GEArray® Human Toxicology and Drug Resistance Microarray (OHS-401; SABiosciences, Frederick, MD). This microarray included 263 key genes that are critical in drug metabolism and resistance, including the encoding enzymes important for drug transport as well as for phase I (specifically the P450 family) and phase II (such as various covalent modification enzymes) metabolism. Target preparation and microarray processing procedures were performed following the manufacturer’s instructions. Briefly, the extracted total RNA was purified with an RNeasy kit (Qiagen, Valencia, CA). Total RNA (20 µg) was used to synthesize double-strand cDNA with SuperScript II reverse transcriptase (Life Technologies Inc., Rockville, MD) and a T7-(dT)24 primer. Then, biotinylated cRNA was synthesized from the double-stranded cDNA using the RNA Transcript Labeling kit (Enzo Life Sciences, Farmingdale, NY), purified, and fragmented. The fragmented cRNA was hybridized to the microarray, which was washed and stained. Fold changes were calculated by comparing transcripts between Tca/PYM-Con and Tca/PYM-siTCRP1 cell lines. A gene was considered differentially expressed when its transcripts showed at least 1.5-fold change.

### Quantitative RT-PCR

Total RNA was extracted and synthesized as described above. The primer sets were synthesized by Invitrogen Biotechnology (Shanghai, China). The expression of seven genes (*MT1X*, *NFκB1*, *EGFR*, *Akt1*, *CYP3A5*, *HIF1A*, and *TP53*) was verified by real-time PCR. The primer sets for PCR amplification were as follows: *MT1X*, forward, 5′-AACTCCTGCTTCTCCTTGCC-3′ reverse, 5′-GCTCTATTTACATCTGAGAGCACAA-3′, *NFκB1* forward 5′- TATTTCAACCACAGATGGCACT-3′, reverse 5′-AGCAAAGGCAATACATACACTT-3′; *EGFR* forward 5′- ATGTTCAATAACTGTGAGGTGGTC-3′, reverse 5′- CAGGAGTAACGGGAGTTGTG-3′; *Akt1* forward5′- GCTGTTCTTCCACCTGTCCC -3′, reverse 5′- CCTGTTCCTGCCCGTGTAAT -3′; *CYP3A5* forward 5′- ATACGGTCATTGCTGTCTCCA -3′, reverse 5′- CTCTTTGAACTCCGCCCTTC -3′; *HIF1* forward 5′- ATTACCCACCGCTGAAACGC -3′, reverse 5′- TCTGTTTCAAGTGGACTCGGATT-3′; and *TP53* forward 5′- GGCCCACTTCACCGTACTAA -3′, reverse 5′- GTGGTTTCAAGGCCAGATGT -3′; Real-time PCRs of these genes were performed according to the standard protocol on a Roche Light-Cycler (Roche, Florence, CA, USA) with SYBR Green detection (TaKaRa SYBR Green Supermix). β-Actin was used as an internal control. The fold change in relative expression of the target gene relative to β-actin was then calculated as described previously [37]. Real-time PCR analysis of all selected genes was repeated thrice.

### Western Blot

Western blot assay was conducted as described previously [7]. Briefly, cells were collected and incubated with cell lysis buffer (0.1 mM NaCl, 0.01 mM Tris–Cl, 1 µM EDTA, 1 µM aprotinin, and 100 µM PMSF) for 20 min at 4°C. Cells were sonicated on ice and centrifuged at 14,000×*g* for 15 min to sediment particulate material. Cell extracts containing equal amounts of protein were mixed with loading buffer and loaded on an SDS–PAGE gel. After electrophoresis, the resolved protein bands were transferred to PVDF membranes and blocked overnight with Tris-buffered saline containing 0.05% Tween-20 (TBST) and 50 g/L nonfat milk at 4°C. The level of proteins was assayed using anti-TCRP1 serum from immunized mouse (1∶2,000) or other antibodies for 2 h at room temperature. After washing with TBST, the cells were incubated with secondary antibody conjugated with peroxidase. The signal was then detected using the chemiluminescent detection system as described by the manufacturer (Amersham Biosciences).

### Immunohistochemical Analysis

Fresh specimens of primary oral squamous cell carcinoma were obtained from the Xiangya hospital. This study was approved by the ethics committee of Xiangya School of Medicine, Central South University, China. For each sample, DDP sensitivity was evaluated using an MTS assay as described previously [7]. A total of 60 samples qualified for inclusion in the final analysis of DDP sensitivity assay, with 30 samples in each group. Immunohistochemical staining was performed on formalin-fixed, paraffin-embedded, 4-µm-thick tissue sections, using the standard SP method. Anti-MT1X antibody with a dilution of 1∶700 was used as the primary antibody. The staining intensity in each section was evaluated by three pathologists and graded according to the average percentage of positive cells in ten randomly selected fields: – (0–5% cells stained),+(5%–20% cells stained),++(20%–50% cells stained), and+++(>50% cells stained).

### Co-immunoprecipitation

Co-immunoprecipitation assay was performed as we described previously [37]. Briefly, the cells were lysed with modified TNE buffer (50 mM Tris [pH 8.0], 150 mM NaCl, 1% Nonidet P-40, 10 mM sodium fluoride, 10 mM sodium pyrophosphate, and 2 mM EDTA) supplemented with 1 mg/l leupeptin, 1 mg/l aprotinin, and 1 mM sodium orthovanadate (Na_3_VO_4_). Immunoprecipitations were performed overnight at 4°C with antibodies to TCRP1 or IgG (as a control). The immunoprecipitates were then incubated for 2 h with protein G-agarose (Amersham Biosciences). The reaction products were washed with lysis buffer, and the immune complexes were resolved by SDS–PAGE. Western blot analyses were subsequently performed.

### Pull-down Assay

Cloning, expression and purification of recombinant GST-TCRP1 protein using a Glutathione Sepharose 4B column (GE Healthcare) was done as described previously [7]. For pull-down assay, ten micrograms of GST or GST-tagged TCRP1 were incubated with Tca8113/PYM cell lysates for 4 h at 4°C. The precipitated proteins were eluted by adding 2×SDS sample buffer (Takara) and detected by immunoblotting with antibodies against GST, Akt or MT1X.

### Cytotoxicity Assay, Cell Cycle Analysis and Colony Forming Assay

For MTS assay, various cells were seeded at 3×10^3^ cells/ml per well in a 96-well plate. After 8 h, the cells were exposed to DDP or PYM (Shanghai Pharmacy Co., China) for 48 h, and relative cell growth was assessed by staining with MTS for 4 h at 37°C according to the manufacturer’s instructions. The plates were read at 490 nm in a Microplate Reader model 450 (Bio-Rad Instruments, USA) to obtain absorbance values. Cellular proliferation in the presence of DDP or PYM was compared with untreated samples. Cell cycle analysis was performed by flow cytometry after propidium iodide (PI) staining using standard protocol as we described previously [6]. All experiments were repeated thrice. For colony-forming assay, Tca/PYM cells were transfected with siRNA against MT1X for 48 h, then incubated under various concentrations of DDP for 24 h (followed by 12 days of incubation in drug-free media).

### Statistics

Data are expressed as mean ± standard deviation. Statistical analyses were conducted with SPSS for Windows, Version 11.0 (Chicago, IL, USA). Student’s *t*-test was used to evaluate statistical significance. Mann–Whitney U test was used to compare the differences in TCRP1 and MT1X immunoreactivity with varying degrees of immunohistochemical staining scores (−, +, ++, +++) between the resistant and sensitive groups. Spearman's correlation coefficient was used to analyze whether the expression patterns of TCRP1 and MT1X in primary tumors correlated with their drug resistance patterns. *P*<0.05 was considered statistically significant.
